# Genomics of the OLIG family of a bHLH transcription factor associated with oligo dendrogenesis

**DOI:** 10.6026/97320630015430

**Published:** 2019-06-15

**Authors:** Shouhartha Choudhury

**Affiliations:** 1 Department of Biotechnology, Assam University, Silchar 788011, Assam, India

**Keywords:** OLIG family, bHLH transcription factor, oligo dendrogenesis

## Abstract

The glial cell neoplasms are not fully classified by using cellular morphology. However, this is possible using known molecular markers in glial development. Oligo-dendrocyte lineage gene induces differentiation of neural progenitors and putative immature progenitor cells of the adult central nervous system. These oligo-dendrocyte lineage genes OLIG1 and OLIG2 encode basic helix-loop-helix transcription factors. The murine bHLH transcription factors found in chromosome 21 are essential for oligo-dendrocyte development. Moreover, OLIG3 of the OLIG family is known to be linked with the brain and spinal cord development. Therefore, it is of interest to analyse oligodendrocyte lineage genes in the OLIG family of bHLH domain for the understanding of oligo-dendrogenesis in eukaryotes. Several bHLH domain linked basic-helix-loop-helix transcription factors in Homo sapiens and Mus musculus from this analysis are reported. Thus, genomics data analysis of OLIG family of bHLH transcription factors help explain observed similarity and differences within the molecular evolutionary context and hence assess the functional significance of the distinct genetic blueprints

## Background

The primary tumors of the human brain are thought to be glial cell origin. The glial cell neoplasm cannot be fully classified by cellular morphology or conventional markers for astrocyte or oligodendrocyte. The diagnostic potential OLIG markers identified oligodendroglial tumors. The oligodendrocyte lineage transcription factors originally identified in rodent encoded bHLH transcription factors. In a rodent central nervous system, they are exclusively expressed in oligodendrocyte. OLIG1 promote the formation of chondroitin sulfate proteoglycan-positive glial progenitors. It is suggested that novel molecular markers are found among factors that have roles in glial development. The markers for different types of cells were known and it is stated that Albert Einstein's brain contains significantly more glia than normal brains in the left angular gyrus 1. The human OLIG1 and OLIG2 express strongly in oligodendroglioma with contrasting low expression in the astrocytoma. These studies show that neoplastic cells of oligodendroglioma resemble oligodendrocyte derived from cells of this lineage. The OLIG1 gene mapped to chromosome 21q22.11 base on sequence alignment in genomic data has roles in the development and maturation of oligodendrocytes especially within the brain. The OLIG1 have an essential role in oligodendrocyte differentiation and consequent remyelination. OLIG1 exhibited failure of remyelination induces lesions and contrasting extensive remyelination of normal controls. A genetic requirement for OLIG1 is repairing the types of lesions occurring in patients with multiple sclerosis 2-7. OLIG2 is essential for the oligodendrocyte and motoneuron development in the spinal cord. OLIG2 encoded BHLHB1 deduced 357 amino acids region has bHLH domain characteristic of transcriptional regulators. OLIG2 positive cells in the late fetal telencephalon primarily develop into astrocyte. OLIG2 expression was upregulated in neoplastic oligodendrocyte yet not in neoplastic astrocyte in brain tumor cells inferring its specific marker of oligodendroglial tumors. OLIG2 coexpressed in motoneuron progenitors and differentiation functioning as a transcriptional repressor. It is hypothesized that it represses the expression of target genes repressors of motoneuron differentiation. OLIG2 functioned sequentially motoneuron and oligodendrocyte fate specification was mainly expressed in the nucleus of neural stem cells, neurons and the cytoplasm of the astrocyte. Knockdown of OLIG2 significantly reduced tumorigenicity in a murine model of malignant glioma and restored tumorigenic phenotypes showing OLIG2 function was specifically required for glioma formation. OLIG2 bound and repressed the expression of p21 in the inhibitor of the cell cycle 8-14. The basic helix-loop-helix, oligodendrocyte transcription factor 2 regulates the fate of a neuron, astrocyte and the oligo-dendrocytes. These factors are co-expressed in neural cells shown by time-lapse imaging and expressed in an oscillatory manner in neural cells. In the differentiation of the lineage, one of the factors becomes dominant 15, 16. Infection of cortex lesion with retroviral vectors containing a dominant form of OLIG2 significantly infected cells generating immature neurons concluded OLIG2 is a repressor of neurogenesis in cells reacting into brain injury. OLIG2 showed normal development of GABAergic neurons and astrocytes in the basal forebrain area. The OLIG3 is the third member of OLIG family found to the chromosome 6q23.3 base on the alignment of sequence in genomic data and play role in the development of 'class A' and 'class B' neurons. OLIG3 is expressed in neural progenitor cells in embryonic and quickly down regulated in post mitotic neurons of the dorsal spinal cord. OLIG3 mutant impaired development of 'class A' neurons; dI1 neurons were generated to reduce numbers, and dI2 and dI3 neurons misspecified and assumed the identity of 'class B' neurons. Conversely, OLIG3 represses the emergence of 'class B' neurons in spinal cord development. OLIG3 distinguishes major classes of progenitors in the dorsal spinal cord and determine distinct specification program of 'class A' neurons 8, 16-20.

The bHLH (basic helix-loop-helix), one of the largest transcription factors containing protein structural motif is characterized by two a-helices connected by a loop. The bHLH domains contain helix motif to bind specific DNA. The bHLH TFs is dimeric with specific DNA binding functions. The basic helix-loop-helix is conserved and characterizes the largest families of transcription factors in eukaryotes. The bHLH transcription factors are made of 40-50 amino acids with two amphipathic alpha helices separated by a linker region. The peptide sequence in the bHLH domain has specific motifs for binding to DNA sequence 21-25. Thus, the transcription factors are the key regulatory proteins to bind specific DNA sequences 26. The function of the transcription factor is regulated in the cell. Transcription factors are one of the groups of proteins, which read and interprets genetic 'blueprint' in DNA. They bind to the DNA and initiate increase or decrease of gene transcription. By turning gene transcription on or off in a cell, transcription factors play roles in development and disease response. Groups of transcription factors in a coordinated fashion direct cell division, cell growth and, cell death. There are approximately 2600 proteins in the human genome containing DNA-binding domains with presumed function as transcription factors 27-29. During embryonic development, many transcription factors contribute to complex morphogenesis in animal development. The identification of numerous gene families across the vast known literature in 'Mus musculus' models help develops transcription factor datasets. Therefore, it is of interest to manually mine the literature for OLIG family of the bHLH transcription factor in Homo sapiens and Mus musculus with known heterogeneity of development and disease susceptibility of oligo dendrogenesis.

## Methodology

### Primary query sequence database and tools:

Primary query sequence was retrieved from different databases (UniProt, EMBL, GenBank and NCBI). The web based application SMART was used for the identification of specific domain in a given sequence. Pfam was used for retrieving protein family information. PROSITE was used for identification of the domain, family, and functional sites as well as associated pattern and profile. PROCHECK was used to examine the stereo-chemical quality of the primary peptide sequence. The genome sequences were downloaded from genomic data in different specialized databases (NCBI, Ensemble and TIGR).

### Standalone tools and GO annotation:

Domain specific profile search was completed using HMMER. HMMER is a statistical algorithm, making use of multiple sequence alignment (MSA) for specific domains in profile search. It uses a probabilistic model called hidden Markov model. Standalone BLAST was used for searching homologous gene in Homo sapiens and Mus musculus. The BLAST2GO was used for the accurate retrieval of specific sequences in the genome. BLAST2GO is a tool for high-throughput gene annotation of the novel sequence data. Functional information was retrieved using Gene Ontology (GO) annotation, which is a controlled vocabulary of the functional attributes.

### Domain, motif, and phylogeny:

Multiple sequence alignment (MSA) methods were used to calculate the best match for homologous sequences for identities, similarities, and differences between them. MSA of multiple sequence hits was carried out with a web-based tool MultAlin for the identification of conserved bHLH domain. The development of the molecular evolutionary relationship between Homo sapiens and Mus musculus was completed using MEGA7 (a tool for phylogenetic tree using Neighbor-Joining method). The MEME suite is a computational tool for analysis of sequence motifs and thus specific motifs in a given query sequence was retrieved using the MEME web-based Tool.

### Gene expression and chromosome location:

Gene expression analysis was carried out using the GENEVESTIGATOR tool, which is a high-performance search engine for gene expression of different biological events. Chromosome location was retrieved using gene card (a database of the human genes provides genomic, proteomic, transcriptomic genetic and functional information on all known and predicted human genes).

## Results

In this study, a survey of OLIG family of the bHLH transcription factor in Homo sapiens and Mus musculus was completed. High confidence bHLH transcription factor data in both the organisms for the understanding of the development of multi cellular organisms are created.

### Sequence data analysis:

**Figure 1:**
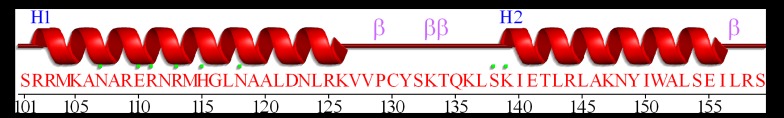
Primary protein sequence of GenBank Id: NM_005806.4

The primary coding region of the gene and its 972 nucleotide translated 323 residues long peptide sequence with 55 residues long binding region to specific DNA sequence is known. The conserved bHLH domain with 55 amino acids is characterized by two a- helices connected by the loop (Figure 1). The flexibility of the loop allows dimerization, folding and packing against another helix, both amphipathic alpha helices separated by a linker region.

### Standalone tools:

The HMMER results gave a total of 59 and 57 bHLH domains. The standalone BLAST results gave a total of 28 and 22 homologous sequences in Homo sapiens and Mus musculus, respectively. The transcription factor data analysis suggested 7 OLIG genes as well as 57, 58 bHLH domains in Homo sapiens and Mus musculus, respectively (Table 1). The OLIG family of the bHLH transcription factor is essential for the functional redundancy of a specific transcription factor in the genome.

### GO annotation:

The gene ontology annotation summary identified the bHLH domain in both the organism's genome (Table 2).

### Multiple sequence alignment:

**Figure 2:**
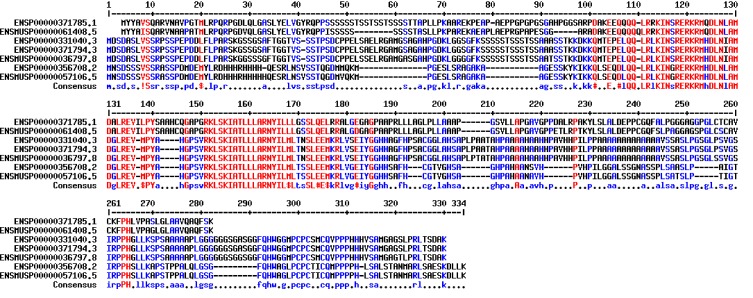
Multiple Sequence Alignment (MSA) of high consensus conserved bHLH domain

**Figure 3:**
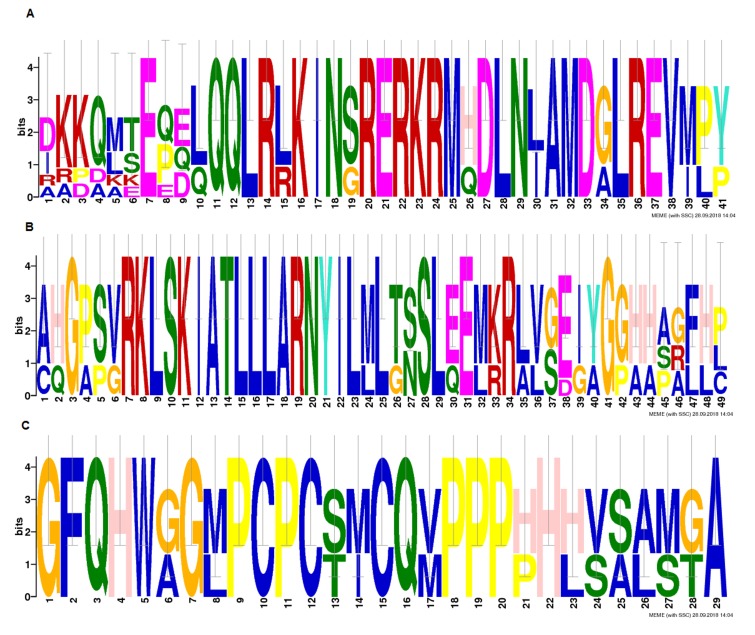
Motifs (a), (b) and (c) in the bHLH domain are shown

Multiple sequence alignment (MSA) result showed conserved domain having high consensus with extended basic helix-loophelix domain (Figure 2) indicating specific motifs (Figure 3).

### Phylogeny:

**Figure 4:**
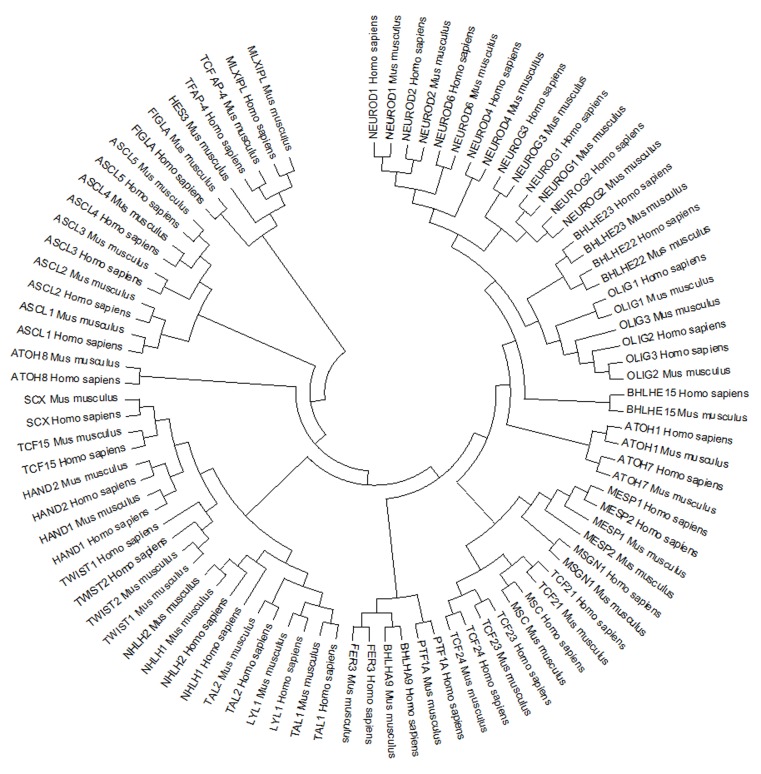
Phylogenetic tree showing evolutionary relationship between Homo sapiens and Mus musculus showing particular clade representing the multifunctional bHLH transcriptional factor gene.

The phylogenetic tree demonstrated the molecular evolutionary relationship between OLIG1, OLIG2, and OLIG3 in Homo sapiens and Mus musculus. Some clades define bHLH domain encoding genes in both human and mouse (Figure 4).

### Gene expression:

**Figure 5:**
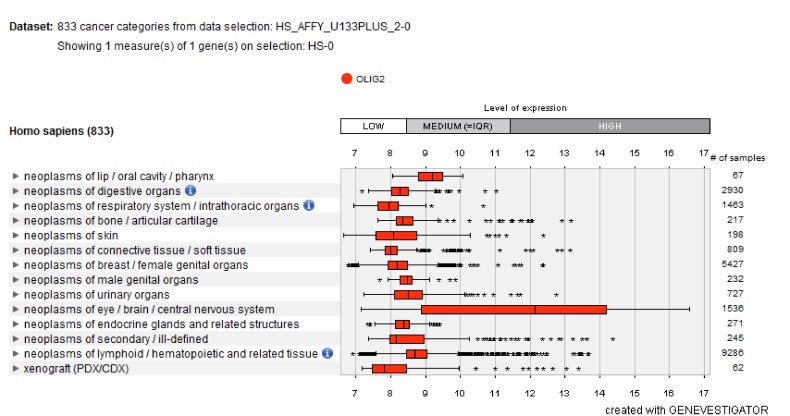
Expression analysis of highly expressed OLIG2 in neoplasm of the eye, brain, and central nervous system

Gene expression analysis of OLIG2 shows that it is highly expressed in neoplasm of the eye, brain, and central nervous system (Figure 5).

### Chromosome location:

**Figure 6:**
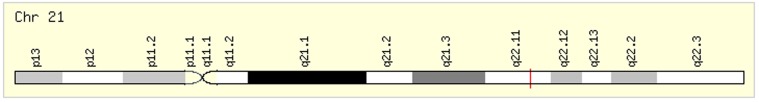
Chromosome location of OLIG2 located band 21q22.11 in Human.

The chromosome localization study shows that OLIG2 is located at band 21q22.11 (Figure 6) in the human. The family-wise classification is essential for a better understanding of the organism's gene contents in this context. Thus, the genome wide analysis identified the genes OLIG1, OLIG2 and OLIG3 with conserved bHLH domain.

## Discussion

 The salient features of genes OLIG1, OLIG2 and OLIG3 having conserved bHLH domain that are associated with brain tumor are discussed. The astrocytoma, oligodendroglioma, and oligoastrocytoma collectively referred as diffuse glioma is a common primary brain tumor. These data classified similarity to astrocyte and oligodendrocyte. The lineage markers represent close to morphologic classification. The murine bHLH transcription factors express neural progenitor and oligodendroglia essential for oligodendrocyte development. OLIG2 closely restricted to the normal oligodendroglia in the human brain. OLIG2 is highly expressed in diffuse glioma. The specific group of oligodendroglioma is susceptible to adjuvant therapy and it is important to elucidate the biological characteristic of tumors. High expression of OLIG1 and OLIG2 is also seen in anaplastic oligodendroglioma and astrocytoma. The oligodendroglial lineage associated markers OLIG1 and OLIG2 are expressed in different glioma. The expression of OLIG2 enables oligodendroglioma and distinguishes glioblastoma from other astrocytic glial tumors. OLIG2 markers of diffuse glioma are expressed in astrocytoma preclude glioma. OLIG2 participate in transcriptional system governing cell fate specification in the ventral spinal cord. Selective interaction of OLIG2 directed motor neuron fate further promoting oligodendrocyte production. The oligodendroglial marker OLIG2 is universally expressed in diffuse glioma and lower in other brain tumors. OLIG1 and OLIG2 help to define the real spectrum of oligodendroglial tumors, which may include a wide variety of tumors with different prognoses. The combinatorial interaction between pro-neural transcription factor NEUROG2 involved in the genesis of motor neuron and oligodendrocyte is seen.

Genetic markers and particularly the loss of 1p and 19q chromosomes have been predicted for prognosis and response to treatment. These emerging techniques will be very helpful in the clinical practice for refining classification and as a therapeutic indication of the oligodendroglial tumors 30-35. OLIG3 is a third member of OLIG family of bHLH transcription factor coordinate specification of the dorsal neuron in the spinal cord. The dorsal horn neurons integrate and relay sensory information and arise during development in the dorsal spinal cord and alar plate. The class A and B neurons emerge in dorsal and ventral alar plate and dependence on roof plate signals for specification and settle in the deep superficial dorsal horn. The OLIG3 in progenitor cells generate 'class A' (dI1-dI3) neurons that is important in the development of neuronal cell types. OLIG3 mutant development of 'class A' neuron; dI1 neurons generally reduced the number, whereas dI2 and dI3 neurons are misspecified and assume the identity of class B neurons. Conversely, OLIG3 repress emergence of class B neurons in the spinal cord. OLIG3 was transiently expressed in lateral margin of sub-ventricular zones as three ventral clusters at the level of p3, p2, and p0 domain in the dorsal neural tube. OLIG3 is expressed in different types of progenitors in the embryonic central nervous system and disappear in course of development. OLIG3 was first detected in the dorsal neural tube from midbrain/hindbrain and spinal cord. Those results suggest that OLIG3 distinguishes major classes of progenitors in the dorsal spinal cord and determines a distinct specification program of 'class A' neurons 20, 36, 37. In this study, information of OLIG family of the bHLH transcription factor in eukaryotes is discussed using data from available databases and published contents.

## Conclusion

The OLIG family of bHLH domain is associated with oligodendrogenesis in eukaryotes. Several bHLH domain linked basichelix- loop-helix transcription factors in Homo sapiens and Mus musculus from literature are discussed. Genomics data analyses of OLIG family of bHLH transcription factor is helpful to characterize gene contents to explain observed similarity and differences within the molecular evolutionary context and hence assess the functional significance of the distinct genetic blueprints.

## Conflict of Interest

The author did not avail of any financial assistance from any source in undertaking the present study. The author declares that there is no conflict of interests regarding the publication of this article.

## Figures and Tables

**Table 1:** Summary of the OLIG family of TF�s

Gene	Homo sapiens	Mus musculus	Total
OLIG1	1	1	2
OLIG2	2	1	3
OLIG3	1	1	2
BHLHE23	2	1	3
BHLHE22	1	1	2
NEUROD1	1	1	2
NEUROD2	1	1	2
NEUROD4	1	1	2
NEUROD6	1	1	2
NEUROG1	1	1	2
NEUROG2	1	1	2
NEUROG3	1	1	2
BHLHA15	2	1	3
ATOH1	1	1	2
ATOH7	1	1	2
ATOH8	1	1	2
FER3DL	1	1	2
SCX	1	1	2
TCF15	1	1	2
HAND1	1	2	3
HAND2	2	1	3
PTF1A	1	1	2
TWIST1	2	2	4
TWIST2	2	2	4
ASCL1	1	1	2
ASCL2	1	2	3
ASCL3	1	1	2
ASCL4	1	1	2
ASCL5	2	1	3
TAL1	2	3	5
TAL2	1	1	2
NHLH1	1	1	2
NHLH2	2	3	5
TCF21	2	2	4
MSC	1	1	2
TCF24	1	1	2
MESP1	1	1	2
MESP2	1	1	2
BHLHA9	1	1	2
LYL1	1	1	2
TCF23	1	1	2
FIGLA	1	1	2
TFAP4	1	2	3
MSGN1	1	1	2
MLXIPL	3	2	5
HES3	0	2	2
Total	57	58	115

**Table 2:** GO annotation summary in the complete genome

Gene Id	Protein
	Homo sapiens
ENSP00000371785.1	oligodendrocyte transcription factor 1
ENSP00000331040.3	oligodendrocyte transcription factor 2
ENSP00000371794.3	oligodendrocyte transcription factor 2
ENSP00000356708.2	oligodendrocyte transcription factor 3
ENSP00000359371.2	class E basic helix-loop-helix protein 23
ENSP00000480998.1	class E basic helix-loop-helix protein 23
ENSP00000318799.1	class E basic helix-loop-helix protein 22
ENSP00000242994.3	neurogenic differentiation factor 4
ENSP00000326391.2	class A basic helix-loop-helix protein 15
ENSP00000476312.1	class A basic helix-loop-helix protein 15
ENSP00000306754.4	neurogenic differentiation factor 2
ENSP00000317333.3	neurogenin-2
ENSP00000295108.3	neurogenic differentiation factor 1
ENSP00000297142.3	neurogenic differentiation factor 6
ENSP00000242462.4	neurogenin-3
ENSP00000317580.4	neurogenin-1
ENSP00000362777.3	protein atonal homolog 7
ENSP00000302216.3	protein atonal homolog 1
ENSP00000275461.3	fer3-like protein
ENSP00000476384.1	basic helix-loop-helix transcription factor scleraxis
ENSP00000304676.3	protein atonal homolog 8 isoform X2
ENSP00000246080.3	transcription factor 15
ENSP00000352565.4	dHand protein
ENSP00000231121.2	Heart and neural crest derivatives expressed 1
ENSP00000365687.3	pancreas transcription factor 1 subunit alpha
ENSP00000346582.5	twist-related protein 1
ENSP00000477638.1	HAND2 isoform 3
ENSP00000405176.2	twist-related protein 2
ENSP00000482581.1	twist-related protein 2
ENSP00000332293.4	achaete-scute homolog 2
ENSP00000242261.5	twist-related protein 1
ENSP00000334547.3	T-cell acute lymphocytic leukemia protein 2
ENSP00000302189.5	helix-loop-helix protein 1
ENSP00000322087.3	helix-loop-helix protein 2
ENSP00000358519.1	helix-loop-helix protein 2
ENSP00000266744.3	achaete-scute homolog 1
ENSP00000237316.3	transcription factor 21
ENSP00000356857.4	transcription factor 21
ENSP00000321445.4	musculin
ENSP00000455444.1	transcription factor 24
ENSP00000300057.4	mesoderm posterior protein 1
ENSP00000342392.3	mesoderm posterior protein 2
ENSP00000375248.1	class A basic helix-loop-helix protein 9
ENSP00000264824.3	protein lyl-1
ENSP00000294339.3	T-cell acute lymphocytic leukemia protein 1 isoform X1
ENSP00000360951.1	T-cell acute lymphocytic leukemia protein 1 isoform X1
ENSP00000296096.5	transcription factor 23
ENSP00000333097.6	factor in the germline alpha
ENSP00000204517.6	transcription factor AP-4
ENSP00000281047.3	mesogenin-1
ENSP00000435770.1	achaete-scute homolog 3
ENSP00000345420.4	achaete-scute homolog 4
ENSP00000392636.1	carbohydrate-responsive element-binding protein isoform X5
ENSP00000469019.2	achaete-scute homolog 5
ENSP00000472681.1	achaete-scute homolog 5
ENSP00000412330.2	carbohydrate-responsive element-binding protein isoform X2
ENSP00000320886.3	carbohydrate-responsive element-binding protein isoform X1
	Mus musculus
ENSMUSP00000061408.5	oligodendrocyte transcription factor 1
ENSMUSP00000036797.8	oligodendrocyte transcription factor 2
ENSMUSP00000057106.5	oligodendrocyte transcription factor 3
ENSMUSP00000104506.1	class E basic helix-loop-helix protein 23
ENSMUSP00000026120.6	class E basic helix-loop-helix protein 22
ENSMUSP00000051379.3	neurogenic differentiation factor 4
ENSMUSP00000055493.7	class A basic helix-loop-helix protein 15
ENSMUSP00000041373.6	neurogenic differentiation factor 2
ENSMUSP00000029587.7	neurogenin-2
ENSMUSP00000040364.4	neurogenic differentiation factor 1
ENSMUSP00000047016.8	neurogenic differentiation factor 6
ENSMUSP00000054054.1	neurogenin-3
ENSMUSP00000050484.4	neurogenin-1
ENSMUSP00000039801.3	protein atonal homolog 7
ENSMUSP00000098903.4	protein atonal homolog 1
ENSMUSP00000058994.3	fer3-like protein
ENSMUSP00000086511.5	transcription factor 15
ENSMUSP00000043668.7	basic helix-loop-helix transcription factor scleraxis
ENSMUSP00000036981.7	protein atonal homolog 8
ENSMUSP00000044983.3	dHand protein
ENSMUSP00000046999.2	heart and neural crest derivatives expressed transcript 1
ENSMUSP00000124951.2	heart and neural crest derivatives expressed transcript 1
ENSMUSP00000028068.2	pancreas transcription factor 1 subunit alpha
ENSMUSP00000007949.3	twist-related protein 2
ENSMUSP00000139531.1	twist-related protein 2
ENSMUSP00000040089.5	twist-related protein 1
ENSMUSP00000113012.1	achaete-scute homolog 2
ENSMUSP00000009392.4	achaete-scute homolog 2
ENSMUSP00000030124.3	T-cell acute lymphocytic leukemia protein 2
ENSMUSP00000057489.3	helix-loop-helix protein 1
ENSMUSP00000064355.4	helix-loop-helix protein 2
ENSMUSP00000142746.1	helix-loop-helix protein 2
ENSMUSP00000143362.1	helix-loop-helix protein 2
ENSMUSP00000020243.7	achaete-scute homolog 1
ENSMUSP00000027062.5	musculin
ENSMUSP00000151767.1	transcription factor 21
ENSMUSP00000032760.5	mesoderm posterior protein 1
ENSMUSP00000053178.7	transcription factor 21
ENSMUSP00000138827.1	transcription factor 24
ENSMUSP00000050516.1	class A basic helix-loop-helix protein 9
ENSMUSP00000103017.1	mesoderm posterior protein 2
ENSMUSP00000046010.4	protein lyl-1
ENSMUSP00000032070.3	factor in the germline alpha
ENSMUSP00000030489.2	T-cell acute lymphocytic leukemia protein 1 isoform X1
ENSMUSP00000124983.1	T-cell acute lymphocytic leukemia protein 1 isoform X1
ENSMUSP00000125202.1	T-cell acute lymphocytic leukemia protein 1 isoform X1
ENSMUSP00000006818.2	transcription factor 23
ENSMUSP00000155803.1	transcription factor AP-4 isoform X3
ENSMUSP00000005862.7	transcription factor AP-4
ENSMUSP00000116358.1	carbohydrate-responsive element-binding protein
ENSMUSP00000055001.1	mesogenin-1
ENSMUSP00000037702.1	achaete-scute homolog 3
ENSMUSP00000137650.1	achaete-scute homolog 4
ENSMUSP00000092006.1	transcription factor HES-3
ENSMUSP00000137746.1	achaete-scute homolog 5
ENSMUSP00000005507.3	carbohydrate-responsive element-binding protein isoform X2
ENSMUSP00000151815.1	transcription factor HES-3
